# Supplementary material for: Causal Relationship between Aging and Anorexia Nervosa: A White-Matter-Microstructure-Mediated Mendelian Randomization Analysis
Source: Biomedicines. 2024 Aug 16;12(8):1874. doi: 10.3390/biomedicines12081874 (PMC11351342; doi:10.3390/biomedicines12081874)
Supplement: Supplementary file 1 [file biomedicines-12-01874-s001.zip › Supplementary Figure/Figure S8/Leaveoneout plot of MR analysis for TL to ubm-b-1539.pdf]

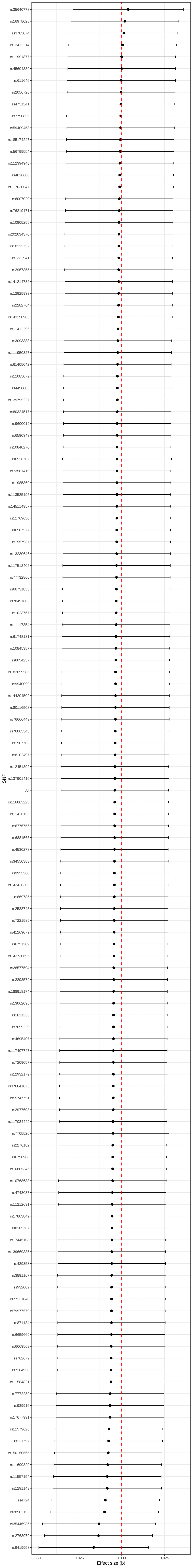
rs35640778
rs16978028
rs3785074
rs12412214
rs11991877
rs45604339
rs611646
rs2056726
rs4731541
rs7790856
rs59409453
rs185174247
rs56799554
rs112394943
rs4616688
rs117630647
rs6007020
rs76219171
rs10905255
rs202034370
rs10112752
rs1332941
rs2967355
rs141214782
rs12925933
rs2282764
rs143190905
rs11412296
rs3093888
rs111950327
rs61405042
rs11085072
rs4498805
rs139795227
rs80324517
rs9600019
rs6590343
rs10840270
rs6536702
rs73581419
rs1985369
rs113525195
rs145114957
rs11769630
rs6587577
rs1957937
rs13230646
rs117512405
rs77732066
rs66731853
rs78491606
rs1023767
rs11117354
rs61748181
rs10845387
rs6054257
rs182059586
rs9940099
rs144204502
rs80116508
rs76666449
rs76065543
rs1907702
rs8102497
rs12451892
rs137901416
All
rs116863223
rs11426156
rs6776756
rs6881568
rs4530278
rs34550383
rs9955360
rs142426306
rs869785
rs2538745
rs7221585
rs41269079
rs6751209
rs142730696
rs28577594
rs2293579
rs188918174
rs13062095
rs1611236
rs7099229
rs4695407
rs117407747
rs7209057
rs12932179
rs376641875
rs55747751
rs2977608
rs117034449
rs7705526
rs2276182
rs6790988
rs10805346
rs10768683
rs4743037
rs11212631
rs17803849
rs8105767
rs17445108
rs139669835
rs429358
rs3891167
rs932002
rs77231040
rs79977579
rs871134
rs6659669
rs6669563
rs762679
rs7164950
rs11584821
rs7772289
rs939916
rs17677991
rs11579626
rs131797
rs150150565
rs11699829
rs11557154
rs1291143
rs4724
rs28502153
rs35446936
rs2763979
rs9419958
SNP
-0.050
-0.025
0.000
0.025
Effect size (b)
